# Brolucizumab for the Treatment of Diabetic Macular Edema: An Optical Coherence Tomography-Based Analysis

**DOI:** 10.3390/diagnostics14242858

**Published:** 2024-12-19

**Authors:** Marco R. Pastore, Serena Milan, Stefano Gouigoux, Olimpia Colombo, Silvia Rinaldi, Gabriella Cirigliano, Daniele Tognetto

**Affiliations:** Eye Clinic, Department of Medical, Surgical Sciences and Health, University of Trieste, 34127 Trieste, Italy; s.gouigoux@gmail.com (S.G.); colombolimpia@gmail.com (O.C.); silvia_rinaldi@hotmail.com (S.R.); gabriellacirigliano16@gmail.com (G.C.); tognetto@units.it (D.T.)

**Keywords:** optical coherence tomography, retinal diseases, spectral-domain optical coherence tomography, brolucizumab, diabetic macular edema, intravitreal injection

## Abstract

**Objectives**: The objectives of this study were to evaluate the structural and functional outcomes after the loading phase with brolucizumab in switched patients with diabetic macular edema (DME) and to identify potential predictive biomarkers of treatment response. **Methods**: A total of 28 eyes with DME, switched to brolucizumab, were retrospectively reviewed. Main outcomes during the follow-up period, up to 6 weeks after the fifth injection, included changes in best-corrected visual acuity (BCVA), central subfield thickness (CST), macular volume, subfoveal choroidal thickness, intraretinal and subretinal fluid (IRF and SRF), cyst dimension including maximal horizontal cyst diameter (MHCD), maximal vertical cyst diameter (MVCD), width-to-height ratio (WHR), foveal avascular zone (FAZ) dimension, and vessel density (VD). **Results**: At the last follow-up, BCVA was significantly improved (*p* = 0.003). Significant reduction of CST was demonstrated after each injection time point (*p* < 0.05), and a dry macula was detected in 64.3% of patients at the last follow-up. The WHR was 1.23 ± 0.46, and a negative correlation to final CST (*p* < 0.0001) was found. In FAZ and VD analysis, no significant variation was detected. At the last disease activity assessment, the treatment regimen was q12 in 64% of patients. **Conclusions**: Brolucizumab leads to anatomical and functional improvements in switched eyes affected by DME. WHR may represent a predictive biomarker of treatment response.

## 1. Introduction

Diabetic macular edema (DME) constitutes a significant cause of vision loss among diabetic patients [1]. Focal and grid laser photocoagulation represented longstanding treatment modalities. However, they are traditionally associated with several complications, such as visual field loss, scotoma formation, change in color vision and night vision, subretinal fibrosis, and neovascularization [2].

A breakthrough in the landscape of DME therapy occurred in the mid-2000s with the introduction of molecular agents targeting vascular endothelial growth factor (VEGF), a key mediator of retinal vascular permeability and neovascularization. Randomized clinical trials of ranibizumab and aflibercept demonstrated their superiority compared to the conventional approach [1,2].

Despite the efficacy of current anti-VEGF agents, the management of DME remains challenging, and there are still some unmet needs, including the need for rapid fluid resolution and the comprehension of the mechanisms behind variability in individual response. The exploration of alternative treatment options is thus required. Brolucizumab, a humanized single-chain antibody fragment targeting VEGF, has recently emerged as a promising agent. It was first approved for the treatment of neovascular age-related macular degeneration (nAMD) in February 2020 by the EMA based on phase 3 HAWK and HARRIER clinical trial results [3]. Its clinical indications were extended to the treatment of visual impairment due to DME after the positive opinion of the Committee for Medicinal Products for Human Use (CHMP) adopted in February 2022. Its non-inferiority with respect to aflibercept in terms of visual outcomes was demonstrated by two clinical trials, KESTREL and KITE [4].

Nevertheless, there is still a lack of real-life reports on its effectiveness because of its recent market introduction, and they are mainly based on case series or studies with limited follow-up. Moreover, there is a growing interest in the identification of structural markers of treatment response.

Optical coherence tomography (OCT) is a noninvasive optical imaging technology based on interference between signals from the object under investigation and a local reference signal. Since its first introduction by Huang and colleagues in 1991, its use has disseminated into clinical practice [5].

As for many other retinal diseases, its employment added new insights also in the evaluation of DME, representing a field under continuous evolution. A comprehensive OCT classification system for DME was introduced in 2020 by the European School for Advanced Studies in Ophthalmology (ESASO), demonstrating great clinical meaningfulness and reliability in the prediction of treatment response [6]. The recognition of patterns of treatment response based on structural parameters is of great clinical significance and constitutes a field of continuous research.

This study aims to report our early experience with brolucizumab for the treatment of DME, analyzing the trend of fluid reabsorption and evaluating potential predictive biomarkers of treatment response.

## 2. Materials and Methods

This is a retrospective observational study at the Eye Clinic, University of Trieste, Italy. Patients affected by DME who received intravitreal injections of brolucizumab (IVB), 6 mg, between May 2023 and April 2024 at our institution were reviewed. Only the eyes that were already treated for DME were enrolled. Eligibility criteria were represented by age ≥ 18 years, a diagnosis of type 1 or 2 diabetes mellitus, and the presence of center-involved DME with central subfield thickness (CST) of ≥320 µm on spectral domain OCT (SD-OCT) at baseline. Patients affected by concomitant ophthalmological diseases (e.g., AMD, glaucoma, and chorioretinal diseases) or autoimmune diseases, those with a previous history of intraocular inflammation (IOI), and those who underwent laser macular treatment were excluded. We also exclude patients with an axial length (AL) inferior to 22.5 mm and higher than 26 mm.

Patients were considered poor responders or refractory to previous anti-VEGF therapy and thus switched to brolucizumab when a CST greater than 300 μm or a reduction in CST of less than 10% was detected after at least 5 prior anti-VEGF injections, according to previous literature [7].

Before starting the treatment, all patients underwent complete ophthalmological examinations, including best corrected visual acuity (BCVA) assessment, intraocular pressure measurement, slit-lamp biomicroscopy, SD-OCT, OCT-angiography (OCT-A), and fluorescein angiography (FA). FA was also repeated at the last follow-up. The most recent (≤3 months) glycosylated hemoglobin (HbA1c), the number of previous injections, and the interval between the last injection of other anti-VEGF and W0 were also registered.

All patients received IVB every 6 weeks and completed the loading phase (5 IVB). The day of the first IVB was considered as the baseline follow-up (W0). Patients were followed up 6 weeks after each injection to obtain disease activity assessment. The last follow-up was 6 weeks after the 5th injection (W30).

At baseline, DME was classified according to the ESASO OCT classification as early, moderate, advanced, and severe [6]. The presence of intraretinal fluid (IRF), subretinal fluid (SRF), epiretinal membrane (ERM), and hyperreflective intraretinal foci (HF) was registered accordingly.

Primary outcomes were represented by the variation in BCVA, CST, macular volume (MV), subfoveal choroidal thickness (SCT), IRF and SRF, the percentage of patients with a CST ≤ 280 μm, cyst dimensions, foveal avascular zone (FAZ) dimensions at the level of the superficial and deep capillary plexus (SCP and DCP), and vessel density (VD) at the SCP and DCP. The secondary outcome was the incidence of IOI and the last treatment regimen.

BCVA, FAZ dimension, and VD were recorded at baseline and last follow-up, while all the other parameters were reviewed six weeks after each injection. BCVA was evaluated using an Early Treatment of Diabetic Study (ETDRS) chart at a 4-m distance and reported as ETDRS letters equivalent (where a BCVA of 20/20 was defined as 85 ETDRS letters) for the statistical analysis.

OCT, EDI-OCT, and OCT-A images were assessed using the Heidelberg Spectralis II OCT (Software Version 6.15, Heidelberg Engineering, Heidelberg, Germany).

CST was defined as the average thickness within the 1-millimeter fovea. MV was defined as the sum of all zone volumes within the 6-mmillimeter circle [8]. SCT was measured from the outer portion of the hyperreflective line corresponding to the retinal pigment epithelium (RPE) to the hyperreflective line corresponding to the sclerochoroidal interface with the manual caliper [9].

IRF was defined as a round, minimally reflective space within the neurosensory retina, located in the outer nuclear layer (ONL), inner nuclear layer, or ganglion cell layer. SRF was defined as a shallow elevation of the retina, with an optically clear space between the outer neurosensory retinal surface and the retinal pigment epithelium (RPE) [6,10].

**Figure 1:**
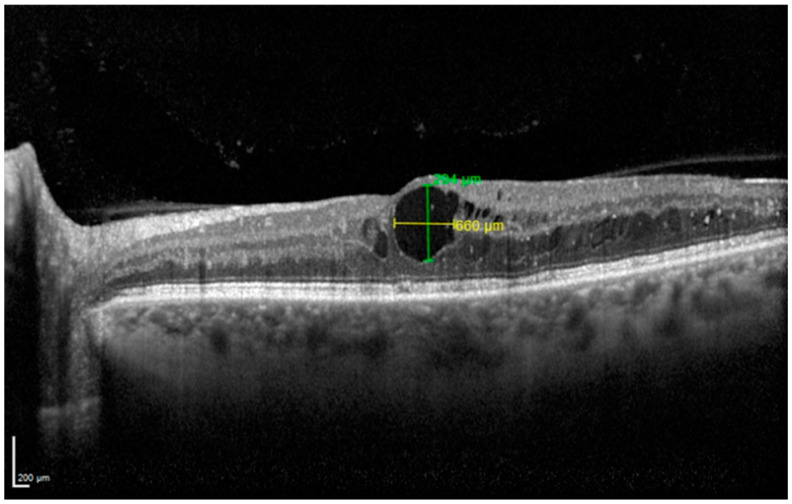
Cyst dimension measurement.

In regard to cyst dimensions, the following parameters were considered: (1) maximal horizontal cyst diameter (MHCD) and (2) maximal vertical cyst diameter (MVCD). These characteristics were analyzed on five horizontal OCT scans: one B-scan passing through the fovea and four B-scans (250 mm and 500 mm superior to the fovea, and 250 mm and 500 mm inferior to the fovea) according to the previous literature (Figure 1) [11].

MHCD and MVCD were used to calculate the width-to-height ratio (WHR).

**Figure 2:**
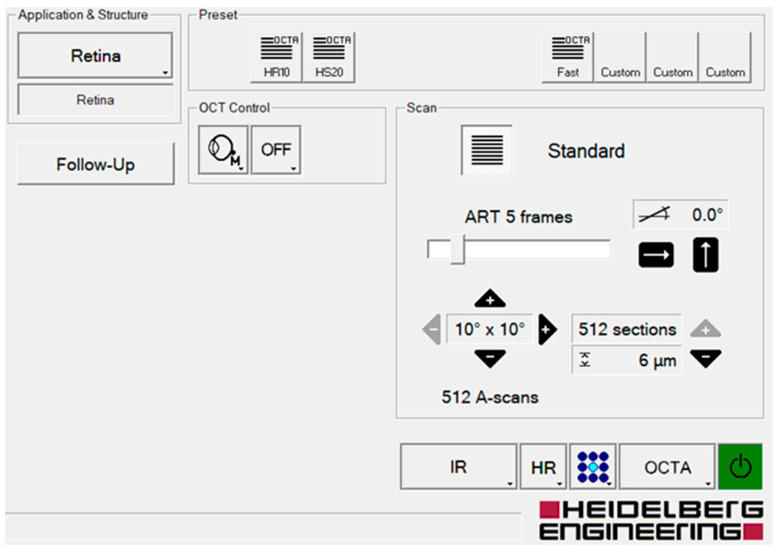
Illustration of the user interface of the Heidelberg Spectralis II OCT-A with the parameters used in this study.

For OCT-A analysis, en-face images were acquired in a high-resolution mode with a 10° × 10° angle and a lateral resolution of 6 μm/pixel, centered on the fovea, resulting in a retinal section of 2.8 mm × 2.8 mm for the visualization of the capillary plexus and an axial resolution of 3.9 microns per pixel yielding precise multilayer segmentation (Figure 2).

Automatic segmentations of the SCP and DCP were carefully reviewed; manual correction was performed in case of errors. FAZ and VD were analyzed by importing enface OCT-A images with their original resolution of 320 × 320 pixels in ImageJ software, version 1.51 (https://imagej.net/ij/; accessed on 23 May 2024; provided in the public domain by the National Institutes of Health, Bethesda, MD, USA) [12].

**Figure 3:**
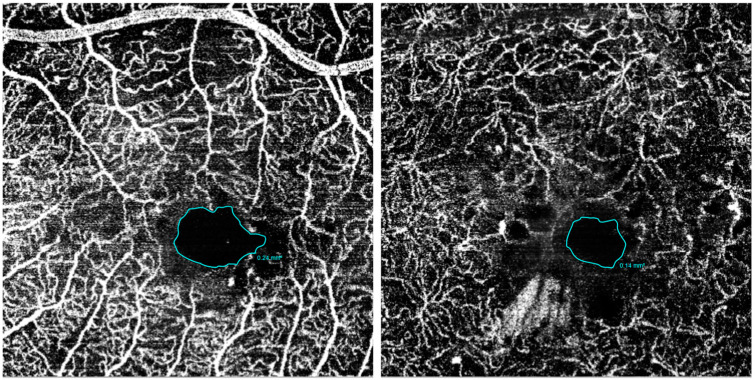
SVP and DVP FAZ measurements.

The FAZ profile was manually delineated using the freehand caliper by interconnecting the most inward-projecting vessel’s ends; the area was then automatically calculated by the software (Figure 3) [13].

**Figure 4:**
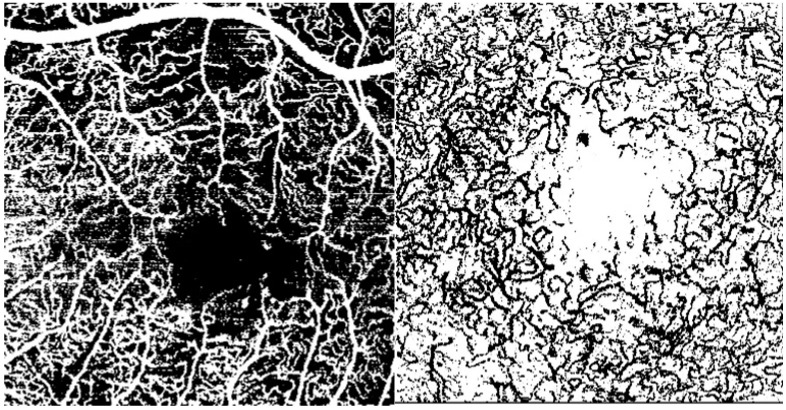
DCP and SVP vessel density after ImageJ processing.

VD was defined as the percentage of the sample area occupied by vessel lumens following binary reconstruction. The original OCTA images of the SCP and the DCP were binarized to convert them from grayscale into black-and-white images using the ImageJ software: each vessel pixel is white while each tissue pixel is black. Capillary VD, defined as the percentage area occupied by the blood vessels, was calculated by counting the number of black pixels and total pixels (Figure 4) [14].

Descriptive analysis was performed using mean and standard deviation (SD) for the continuous variables and percentage values for the categorical variables. A paired *t*-test was used to assess mean differences in CST, MV, and CT between each time point; in FAZ and VD between baseline and first injection; and in BCVA between baseline and last follow-up. Pearson’s correlation coefficient R was analyzed to study the correlation between mean baseline MHCD and final CST, mean baseline MVCD and final CST, and mean WHR and final CST; a corresponding correlation test was used to check the statistical significance.

All statistical analyses were performed using R Statistical Software (version 3.5.3; R Foundation for Statistical Computing, Vienna, Austria). All tests were two-tailed, and a statistical significance was defined as a *p*-value < 0.05.

## 3. Results

Twenty-eight eyes of twenty-eight patients met the inclusion criteria and were included in the analysis. Demographic analysis is reported in Table 1.

Any patient reported glycemic decompensation or any modification in anti-diabetes therapy in the previous year.

**Figure 5:**
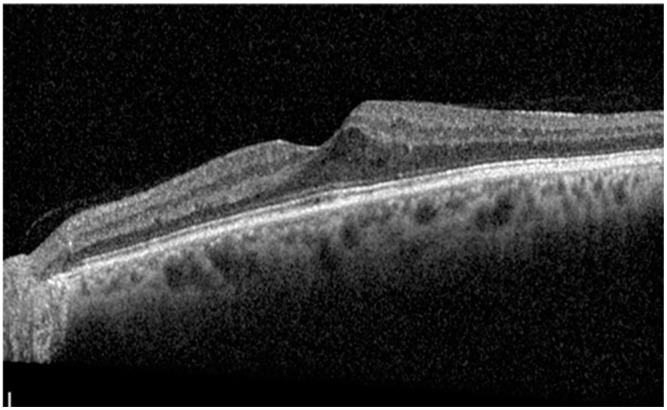
Early diabetic macular edema.

**Figure 6:**
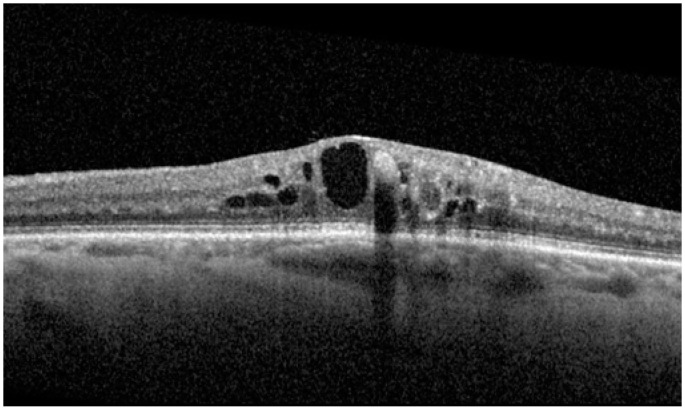
Advanced diabetic macular edema.

**Figure 7:**
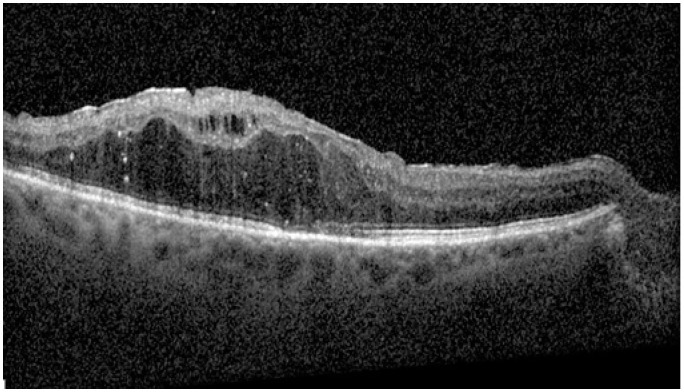
Severe diabetic macular edema.

At baseline, IRF was present in all patients, SRF in any patient, HF in 19 patients (67.9%), and ERM in 12 patients (42.9%). According to DME ESASO Classification, at baseline 4 out of 28 patients (14.3%) were affected by early DME, 20 (71.4%) by advanced DME, and 4 (14.3%) by severe DME (Figure 5, Figure 6 and Figure 7); atrophic maculopathy was not detected in any case [6].

The mean baseline BCVA was 58.93 ± 13.81 letters (20/63). At W30, the mean BCVA was 63.67 ± 14.68 letters (20/50). A significant improvement in BCVA was registered (*p* = 0.003, paired *t*-test), with a mean gain of 4.73 ± 1.78 letters.

CST values at each time point are reported in Table 2.

**Figure 8:**
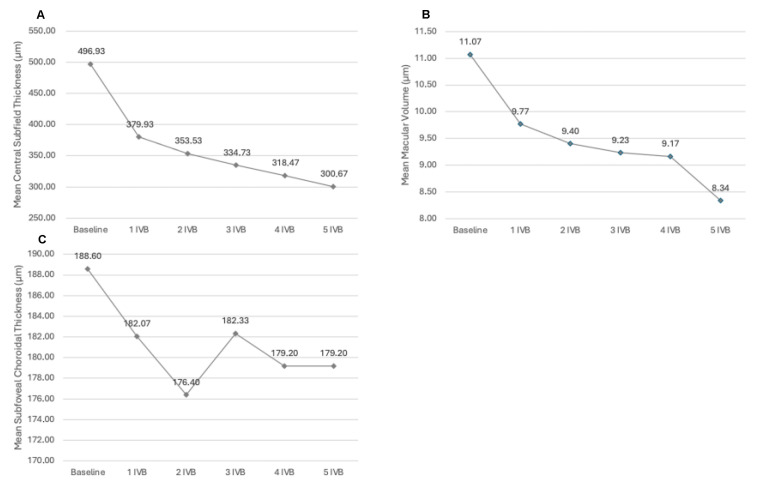
Variations of mean central subfield thickness, mean macular volume, and mean subfoveal choroidal thickness during the intravitreal treatment. Variations of mean central subfield thickness (**A**), mean macular volume (**B**), and mean subfoveal choroidal thickness (**C**) during the intravitreal treatment. Data were recorded 6 weeks after each injection. IVB = intravitreal injection of brolucizumab.

The mean CST variation trend is represented in Figure 8.

A statistically significant reduction in CST was demonstrated when comparing CST values before and 6 weeks after each injection (*p* < 0.05, paired *t*-test). A mean reduction of 169.27 ± 70.35 μm was registered between baseline and last follow-up.

The number of patients with a CST ≤ 280 μm progressively increased after the first injection: at W6 it was detected in 3 (10.7%) patients, at W12 in 9 (32.1%) patients, at W18 in 11 (39.3%) patients, at W24 in 13 (46.4%) patients, and at W30 in 15 (53.6%) patients.

**Figure 9:**
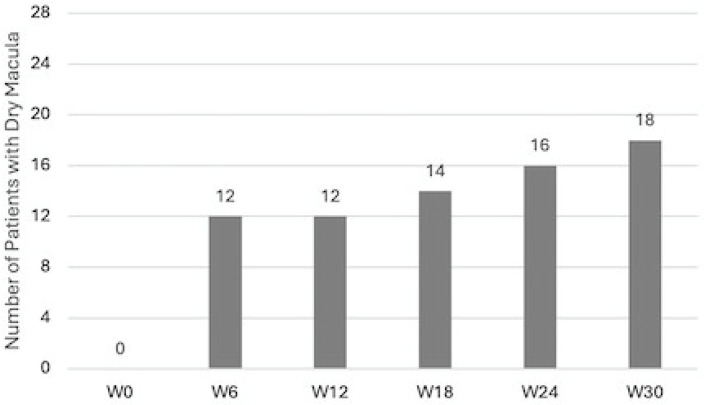
Patients reaching dry macula during treatment with brolucizumab.

SRF was not detected in any patient at any time point. IRF progressively reduced at each timepoint: dry macula was present in 12 (42.9%) patients at W6 and at W12, in 14 (50%) patients at W18, in 16 (57.1%) patients at W24, and in 18 (64.3%) patients at W30. Values are reported in Figure 9.

Patients reaching dry macula during treatment with brolucizumab. Data were recorded 6 weeks after each injection.

MV values at each time point are reported in Table 2. The mean MV variation trend is represented in Figure 8. A statistically significant reduction in MV was demonstrated when comparing MV values before and 6 weeks after each injection (*p* < 0.05, paired *t*-test), except after the second injection (*p* = 0.06). A mean reduction of 2.73 ± 1.56 mm was registered between baseline and the last follow-up.

SCT values at each time point are reported in Table 2 and Figure 8. Any significant result was demonstrated by paired *t*-test.

At baseline, the mean MHCD was 295.4 ± 170.7 μm, and the mean MVCD was 249.3 ± 136.5 μm. The mean baseline WHR was 1.23 ± 0.46. The Pearson correlation test was performed to assess the presence of a correlation between these values and the final CST. WHR and final CST showed a negative correlation (r = −0.65, *p* = 0.0001). No significant correlation was found between MHCD and final CST (*p* = 0.13) and MVCD and final CST (*p* = 0.41).

The analysis of VD and FAZ area is reported in Table 3. No significant result was reported.

IOI was not detected in any patient. The last treatment regimen was q8 in 10 patients and q12 in 18 patients.

## 4. Discussion

In the present study, we report an OCT-based retrospective analysis of the effects of switching from conventional anti-VEGF therapy to brolucizumab for the treatment of DME in a real-life clinical setting.

We demonstrated a prompt control of IRF since the very first injection, with a statistically significant reduction of both CST and MV. These results were maintained at the end of the loading phase. The anatomical restoration was accompanied by functional improvement, as demonstrated by the significant gain in BCVA. Moreover, we outlined a new possible OCT marker of poor response, which is represented by the so-called WHR.

Brolucizumab was approved for the treatment of DME in February 2022 in Europe. The KESTREL and KITE clinical trials showed non-inferiority of brolucizumab to aflibercept in terms of visual outcomes, with a greater proportion of patients achieving central macular thickness (CMT) < 280 μm. However, due to its recent market introduction, real-world results of switching to brolucizumab are still lacking, and they derive from case series or studies with short follow-ups.

The early drying action of brolucizumab in nAMD patients is known, and it seems to characterize also eyes affected by DME [15]. In particular, Hirano et al. demonstrated a significant reduction in central macular thickness (CMT) 4 weeks after a single IVB in 23 DME patients, passing from 536.5 μm to 322.2 μm [16]. Murray et al. reported similar results even after a slightly longer follow-up: they noted a significant reduction in central thickness 6 weeks after a single IVB in 13 DME patients, passing from 418.3 μm to 353.4 μm [17]. Our results are in line with the literature: we noted a significant reduction in CST (passing from 496.93 μm to 379.93 μm) and in MV (passing from 11.07 μm to 9.78 μm) 6 weeks after the first injection.

Two previous studies confirmed brolucizumab efficacy even after multiple IVBS in naïve and switched eyes [18,19]. However, our study is the first to report the results after the completion of the loading phase. We demonstrated a progressively significant reduction of both CST and MV. This means that patients returned for the following treatment presenting a lower amount of IRF compared to the previous visit. We found that at the last follow-up, the proportion of subjects with CST < 280 μm was 53.3%. This is in accordance with KRESTEL and KITE results, in which the proportions were 47.1% and 48.0%, respectively [4].

In our cohort, we also analyzed the modification in IRF. Interestingly, we demonstrated that the first injection was efficient in determining complete reabsorption of fluid in 42.9% of patients. The percentage of patients reaching dry macula progressively increased, ending at 64.3% at W30.

These data confirm the ability of brolucizumab to determine fluid reabsorption even in eyes that are recalcitrant to other treatments. This may be linked to its molecular characteristics. Brolucizumab is characterized by the absence of the Fc domain, a smaller molecular size (26 kDa compared to bevacizumab of 146 kDa, ranibizumab of 48 kDa, and aflibercept of 97–115 kDa), and a great solubility, permitting a concentration of 120 mg/mL [4].

SCT represents another parameter that has been studied in relation to DME. Inconsistent and even contradictory results are reported in the literature, with DME being correlated at times with a thickening of SCT and, at times, with a thinning [20,21,22,23,24]. Intravitreal treatment with both anti-VEGF and steroids has been associated with a reduction in choroidal thickness [25,26]. In our study, SCT didn’t undergo any significant modification. It must be stated that patients were previously treated with intravitreal injections, which probably already determined a shrinkage in choroidal vasculature. In fact, the baseline SCT in our cohort was 188,6 μm. According to the literature, a mean SCT of 230.62 ± 69.57 μm is normally expected in healthy patients in their 70s. A similar result was demonstrated by Tamashiro et al. in nAMD patients: they noted that SCT was significantly lower after IVB, but in switched eyes the decrease was not as marked compared to that of the treatment-naïve group [27].

The anatomical restoration was accompanied by functional improvement, with a final mean gain of 4.73 letters. Greater results were achieved in KESTREL and KITE, in which a mean gain, respectively, of 9.2 letters and 10.6 letters were demonstrated at week 52. However, 73.3% and 13.4% of patients in our cohort were affected, respectively, by an advanced and a severe form of DME according to ESASO classification. Both subtypes can be characterized by an impairment of the ellipsoid zone/external limiting membrane; while in the former it is still visible and inner retinal layer segmentation is generally preserved, in the latter both are mostly undetectable. These alterations can considerably affect visual outcome; thus, our results are in line with this evidence [6].

The understanding of OCT parameters predicting the response to treatment constitutes a field of great clinical importance. Previous studies already outlined different biomarkers associated with an inflammatory pattern of DME and worse functional outcome [28,29]. The significance of the size of ONL cysts has already been investigated. Some authors classified ONL cysts according to their diameter (mild < 100 μm, 100 ≤ moderate < 200 μm, and severe ≥ 200 μm) [30]. However, this kind of classification would have had poor significance in our cohort since 73.3% of the patients included in our analysis presented with severe ONL cysts. We studied the presence of a correlation between baseline cyst diameter and final CST. No significant result was found when analyzing horizontal and vertical diameters. However, we demonstrated a negative correlation between the mean horizontal and vertical diameter ratio (WHR) and the final CST: this means that a greater ratio (determined by cysts that were larger than higher) was associated with a greater drying effect. Accordingly, a higher vertical diameter seems to correlate with a poorer response. This might be due to the anatomical modification induced by this kind of cyst: we hypothesized they determine a stretching effect on the retinal structure, interrupting the Muller cells, which are responsible for retinal integrity. Once these connections are severed, retinal elasticity is compromised, preventing anatomical restorage. We propose the “WHR” as a new possible marker of response to brolucizumab. Other authors analyzed the possible role of the total ONL cystoid area as a prognostic marker. However, this kind of measurement seems unsuitable for routine clinical utilization [31].

The limitations of this study concern the small sample size, as the use of bevacizumab for the treatment of diabetic retinopathy had only recently been approved at the time of data collection. Additionally, especially in cases of severe diabetic retinopathy, obtaining high-quality OCT angiography scans suitable for scientific purposes was challenging due to the altered retinal architecture. Furthermore, only patients who had previously not responded to other anti-VEGF treatments were enrolled in this study, which led to the exclusion of naïve patients and responders to conventional intravitreal therapies for diabetic macular edema.

## 5. Conclusions

In conclusion, our results suggest that brolucizumab may represent an effective option for the treatment of DME. The evaluation of the cyst WHR at baseline could be a useful prognostic indicator for fluid reabsorption: studies with longer follow-up are needed to analyze the maintenance of the result during interval extension after the loading phase.

## Figures and Tables

**Table 1:** Demographic and clinical analysis.

Demographic Data	Results
Number of eyes (patients)	28 (28)
Sex (male/female)	16:12
Age (years), mean ± SD *	69.31 ± 7.63
Mean diabetes duration (years), mean ± SD *	21.2 ± 9.09
Diabetes type (1:2)	4:24
Insulin-dependent (yes/no)	18:10
Last HbA1c (%), mean ± SD *	7.01 ± 0.71
Metabolic decompensation within the last year	0
Proliferative diabetic retinopathy (not active), n of patients	4
Previous intravitreal anti-VEGF ^†^: number of patients	28
Previous intravitreal anti-VEGF ^†^: number of injections (mean ± SD *)	13.3 ± 6.78
Previous intravitreal steroids: number of patients	14
Previous intravitreal steroids: number of injections (mean ± SD *)	3.88 ± 3.96
Interval between the last injection of other anti-VEGF and W0 (weeks) (mean ± SD *)	19.94 ± 12.48

* SD = standard deviation; ^†^ anti-VEGF = antivascular endothelial growth factor.

**Table 2:** Central subfield thickness, macular volume, and subfoveal choroidal thickness variations.

	Week 0	Week 6	*p* *(W6 vs. W0)* *	Week 12	*p* *(W12 vs. W6)* *	Week 18	*p* *(W18 vs. W12)* *	Week 24	*p* *(W24 vs. W18)* *	Week 30	*p* *(W30 vs. W24)* *
Central Subfield Thickness, µm (mean ± SD)	496.93 ± 79.06	379.93 ± 116.96	***0.0001***	353.53 ± 116.11	***0.0081***	334.73 ± 111.99	***0.0017***	318.47 ± 99.58	***0.0073***	300.67 ± 84.61	***0.0017***
Macular Volume, mm^3^ (mean ± SD)	11.07 ± 1.47	9.77 ± 1.72	***0.0089***	9.04 ± 1.82	***0.0682***	9.23 ± 1.85	***0.0365***	9.17 ± 1.88	***0.0247***	8.33 ± 1.48	***0.0114***
Subfoveal Choroidal Thickness, µm (mean ± SD)	188.6 ± 84.67	182.07 ± 89.60	*0.2842*	176.40 ± 83.25	*0.1966*	182.33 ± 80.70	*0.2501*	179.20 ± 81.26	*0.1717*	179.20 ± 81.83	*0.1718*

Variations in central subfield thickness, macular volume, and subfoveal choroidal thickness during treatment with brolucizumab. Mean values ± SD at each follow-up are reported. A paired *t*-test was performed to assess statistical differences before and after each injection. *p* values are reported in italics. Significant results are reported in bold text. SD = standard deviation; W = week; *p* = *p*-value (* paired *t*-test).

**Table 3:** Vessel density and foveal avascular zone area at baseline and after the first injection.

	Baseline	After the 1st Injection	*p*
FAZ * area at the SCP ^†^ (mean, mm^2^ ± SD ^⊥^)	0.33 ± 0.18	0.24 ± 0.04	*0.36*
FAZ * area at the DCP ** (mean, mm^2^ ± SD ^⊥^)	0.36 ± 0.17	0.26 ± 0.04	*0.27*
VD ^‡^ at the SCP ^†^ (mean, % ± SD ^†^)	30.30 ± 0.07	28.34 ± 0.06	*0.14*
VD ^‡^ at the DCP ** (mean, % ± SD ^†^)	21.40 ± 0.06	20.36 ± 0.07	*0.63*

Variations in foveal avascular zone (FAZ) and vessel density (VD) during treatment with brolucizumab. Mean values ± SD at baseline and after the first injection are reported. A paired *t*-test was performed to assess statistical difference between the two timepoints. * FAZ: foveal avascular zone; ^†^ SCP: superficial capillary plexus; ^⊥^ SD: standard deviation; ** DCP: deep capillary plexus; ^‡^ VD: vessel density. *p* values are reported in italics.

## Data Availability

Data are available upon request to the corresponding author.
